# Volatile Organic Compound Emissions in the Invasive Legume *Cytisus scoparius*: Linking Plant Phenology, Arthropod Communities, and Environmental Factors

**DOI:** 10.3390/plants15010095

**Published:** 2025-12-28

**Authors:** Evans Effah, Paul G. Peterson, D. Paul Barrett, Andrea Clavijo McCormick

**Affiliations:** 1 College of Sciences, Massey University, Palmerston North 4472, New Zealand; 2 Bioeconomy Science Institute, Palmerston North 4472, New Zealand

**Keywords:** biological control, chemical ecology, herbivores, pollinators, predators, Scotch broom

## Abstract

Scotch broom (*Cytisus scoparius*; Fabaceae) is an invasive nitrogen-fixing shrub widespread in New Zealand, where it impacts forestry, pasturelands, and native ecosystems. Although several biological control agents have been released, Scotch broom continues to expand in regions such as the North Island’s Central Plateau. Scotch broom affects the germination and growth of other plants and modifies arthropod communities (including pollinators, herbivores, and predators) within its invaded range. Volatile organic compounds (VOCs) play a key role in mediating plant–plant and plant–arthropod interactions, potentially contributing to this invasive plant’s ecological success. However, Scotch broom’s VOC emissions in its invaded ranges remain poorly understood. We examined VOC emissions from flowering and non-flowering Scotch broom plants in the Central Plateau and assessed links with biotic and abiotic factors. Our aims were to (1) characterise differences in VOCs between phenological stages; (2) explore shifts in arthropod community composition; and (3) evaluate correlations between VOC emissions, arthropod groups and environmental variables. Flowering plants had higher diversity and abundance of VOCs, with blends dominated by monoterpenes, aromatics, and fatty acid esters, whereas non-flowering plants were characterised by green leaf volatiles (GLVs). Flowering stages supported Hemiptera and Thysanoptera (herbivores), which were positively correlated with fatty acid esters. In contrast, GLVs correlated with Araneae (predators) abundance. Temperature was the strongest predictor of VOC emission patterns, showing significant correlation with most compound classes. These results advance understanding of Scotch broom invasion ecology and highlight the need to further explore individual compounds potentially influencing arthropod composition to inform both native arthropods conservation and future biocontrol strategies.

## 1. Introduction

Scotch broom *Cytisus scoparius* (L.) Link, (Fabales—Fabaceae) is an early-flowering, nitrogen-fixing shrub, native to western and central Europe. This species is invasive in many temperate regions, including Australia and New Zealand, where it causes serious ecological perturbation and economic losses across plantation forestry, pastoral systems, and native ecosystems [1,2].

Biological control programmes have been undertaken in both countries, using control agents targeting different parts of the plant. These include the stem-feeding broom gall mite (*Aceria genistae* (Nalepa 1891) Trombidiformes: Eriophyidae), broom leaf beetle (*Gonioctena olivacea* (Forster, 1771) Coleoptera: Chrysomelidae), broom psyllid (*Arytainilla spartiophila* (Förster, 1848) Hemiptera: Psyllidae), broom seed beetle (*Bruchidus villosus* (Fabricius 1792) Colepotera: Chrysomelidae), broom shoot moth (*Agonopterix assimilella* (Treitschke, 1832) Lepidoptera: Depressariidae), and the self-introduced broom twig miner (*Leucoptera spartifoliella* (Hübner 1813) Lepidoptera: Lyonetiidae) [3,4,5,6,7]. However, despite anecdotal reports of reduced Scotch broom density in some regions of New Zealand (including Southland), mainly due to the broom gall mite, this species continues to thrive in some areas.

**Figure 1:**
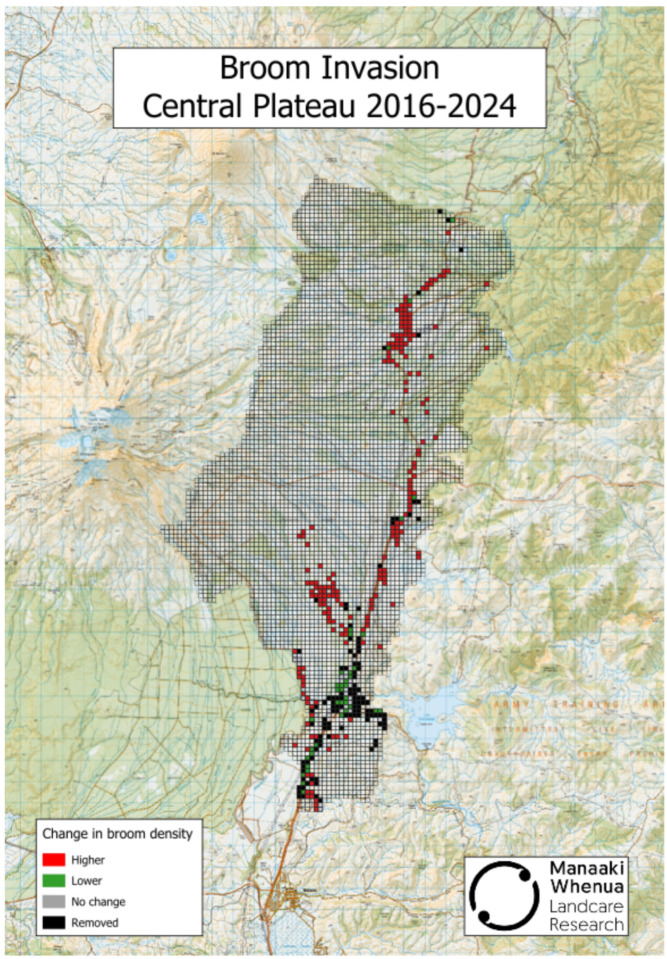
Changes in Scotch broom density following an active herbicide spray programme on the Central Plateau between 2016 and 2024. Data was provided by the Desert Road Invasive Legume Control Programme—Project Yellow|Waikato Regional Council (https://www.waikatoregion.govt.nz/services/biosecurity/project-yellow/, accessed 16 December 2025). Methodology is provided in [8].

In New Zealand, one area where Scotch broom continues spreading despite having an active herbicide spraying programme in place is the Central Plateau of North Island [8], location of the Tongariro National Park, a UNESCO dual World Heritage site (Figure 1). Unchecked, this yellow-flowered nitrogen-fixing legume is likely to affect both soil properties and biota as well as native plant and arthropod communities and the recreational and aesthetic value of the region [9,10,11,12].

The Central Plateau is heavily invaded by another alien species—heather, *Calluna vulgaris* (L.) Hull, (Ericales: Ericaceae)—which has displaced native plants and modified arthropod communities [9,13,14], but a successful biocontrol programme has significantly reduced heather density [15]. Hence, if Scotch broom continues to expand, it may replace heather as the dominant invader due to its rapid growth, profuse flowering, explosive seed dispersal [3,15], and by releasing root exudates that modify soil properties and affect competitors’ seed germination and seedling growth [11,12]. Scotch broom also produces multiple phytochemicals from other plant parts with bioactive properties e.g., herbicidal effects [16,17,18].

Generally, plants are prolific producers of chemical compounds that mediate their interactions with other organisms and contribute to their ecological success [19,20,21,22]. Plants release complex blends of volatile organic compounds (VOCs)—or scents—from flowers and vegetative tissue, which act as signalling molecules or cues that influence the behaviour of pollinators, herbivores (including biocontrol agents), herbivore natural enemies, and neighbouring plants, and can have allelopathic or phytotoxic effects influencing arthropod and plant community composition [23,24,25,26,27].

Empirical studies across plant systems demonstrate that transitions between reproductive (flowering) and vegetative stages can produce large qualitative and quantitative shifts in VOC emissions [28,29,30,31], these shifts can alter plants’ attractiveness to pollinators, herbivores, and plant and insect natural enemies. In the case of invasive shrubs such as heather and Scotch broom, VOC-mediated effects may have ecological and management relevance. For instance, floral emissions that attract pollinators may enhance seed set and invasion potential, while vegetative VOCs that are phytotoxic or that attract predators or parasitoids of herbivores could alter competitive and trophic dynamics in invaded communities [14,17,32]. From a biocontrol perspective, plant VOCs could improve the performance of biocontrol agents by enhancing attraction to their host (plant or insect), promoting their reproduction, and aiding establishment. However, they may also hinder their performance by attracting predators or competitors of the biocontrol agent [33].

Volatile emissions are closely linked to abiotic and biotic factors, which may influence their production, emission, and signalling properties. Notably, temperature, soil nutrients, water content, UV radiation, environmental pollutants such as CO_2_, and interactions with insects and microorganisms are known factors modulating VOC emissions. The underlying mechanisms may vary, including changes in enzymatic activity, cellular structure, transport, and the bioavailability of substrates [34,35,36,37]. In the case of invasive species, it is expected that they will encounter different biotic and abiotic factors from those in their native ranges, leading to metabolic shifts that may alter both constitutive metabolites and VOC emissions and their info-chemical value to native and introduced organisms [38,39,40,41].

While Scotch broom is known to affect arthropod community composition and to produce a range of volatile compounds that vary among plant tissues [12,16,17,18], no study has investigated these factors together in either the plant’s native or invaded ranges. Understanding the relationships between VOCs, plant phenology, arthropod communities, and environmental factors in invasive species such as Scotch broom could be useful for informing biocontrol management strategies. Such insights can help identify specific compounds that influence the behaviour and performance of pollinators and biocontrol agents, as well as the environmental conditions that regulate their emission. This knowledge could also improve our understanding of how invasive plant VOCs influence the behaviour and interactions of native arthropods, supporting their conservation. While these interactions are inherently complex, advances in analytical technologies and data processing are increasingly enabling us to unravel and interpret these intricate ecological networks.

The aim of this paper is to fill this knowledge gap by exploring links between Scotch broom’s VOC emissions, plant phenology (flowering vs. non-flowering), associated arthropod communities, and abiotic factors in New Zealand. Given evidence that (1) broom flowering produces volatile bioactive blends, (2) floral vs. vegetative VOCs have distinct ecological functions, and (3) biotic and abiotic factors influence VOC emissions, we hypothesised that the transition between flowering and non-flowering stages (and associated environmental changes) will substantially change Scotch broom’s volatile emissions, attract different arthropod taxa, and ultimately modify info-chemical networks in invaded sites. The overarching goal of this study is to support Scotch broom management by advancing our understanding of the species’ chemical ecology, thereby enabling the future development of tools to enhance biocontrol strategies.

## 2. Results

### 2.1. Volatile Emissions

**Figure 2:**
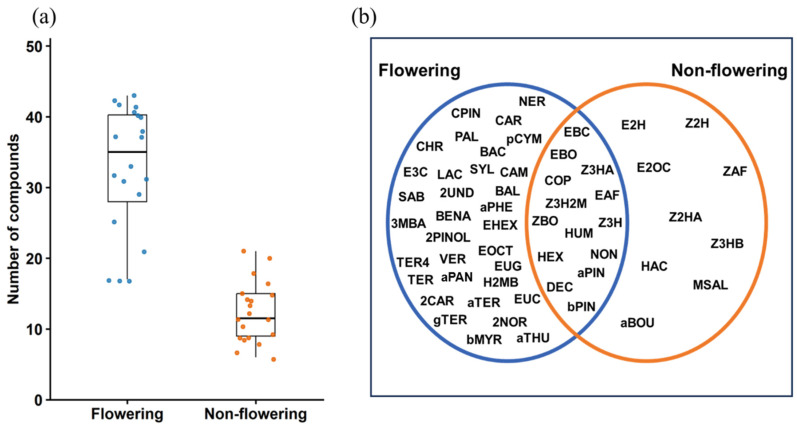
(**a**) Total number of compounds identified in the Scotch broom headspace. (**b**) Venn diagram showing compounds identified exclusively from flowering plants, exclusively from non-flowering plants, and those shared between both phenological stages. Compound codes correspond to those listed in Table 1.

A total of 56 VOCs were found in the headspace of Scotch broom (Table 1). Flowering and non-flowering plants differed in their VOC composition, with flowering plants releasing a significantly higher number of compounds (Wilcoxon rank-sum test: W = 390.5, *p* < 0.001). Some compounds were detected exclusively in either flowering or non-flowering plants, whereas others were found in both, with varying abundance (Table S1). Flowering plant emissions were characterised by terpenes, aromatic compounds and fatty acid esters, while non-flowering plants were characterised by green leaf volatiles (Figure 2a,b).

**Figure 3:**
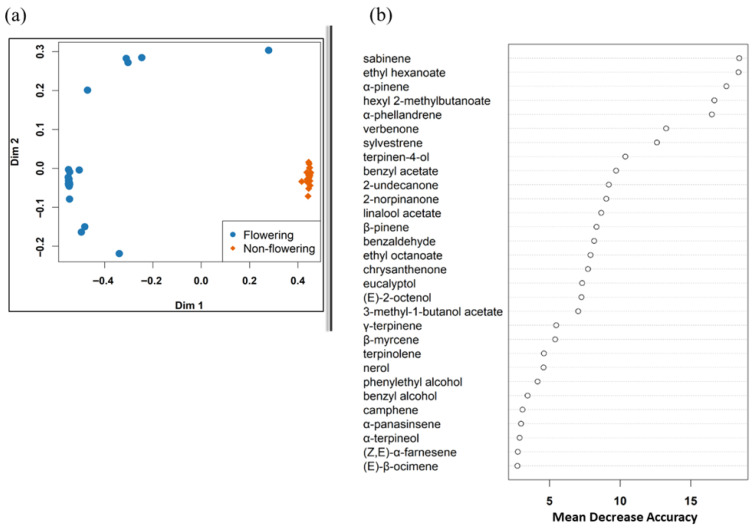
(**a**) Multidimensional scaling (MDS) plot showing how samples were clustered based on identified compounds. (**b**) Top 30 VOCs that were important in distinguishing between volatile blends of flowering and non-flowering Scotch broom plants.

A random forest model correctly classified 19/20 flowering plants and 20/20 non-flowering plants, with an overall accuracy of 97.5% (OOB 2.5%). Sabinene, ethyl hexanoate, α-pinene, hexyl 2-methylbutanoate, α-phellandrene, verbenone, sylvestrene, terpinen-4-ol, benzyl acetate, and 2-undecanone were found to be the ten most important compounds for distinguishing the blend of flowering and non-flowering plants (Figure 3). These were all emitted in higher amounts by flowering plants and virtually absent in non-flowering ones. Of these, α-pinene, sabinene, and α-phellandrene were the most abundant (Table 2).

**Figure 4:**
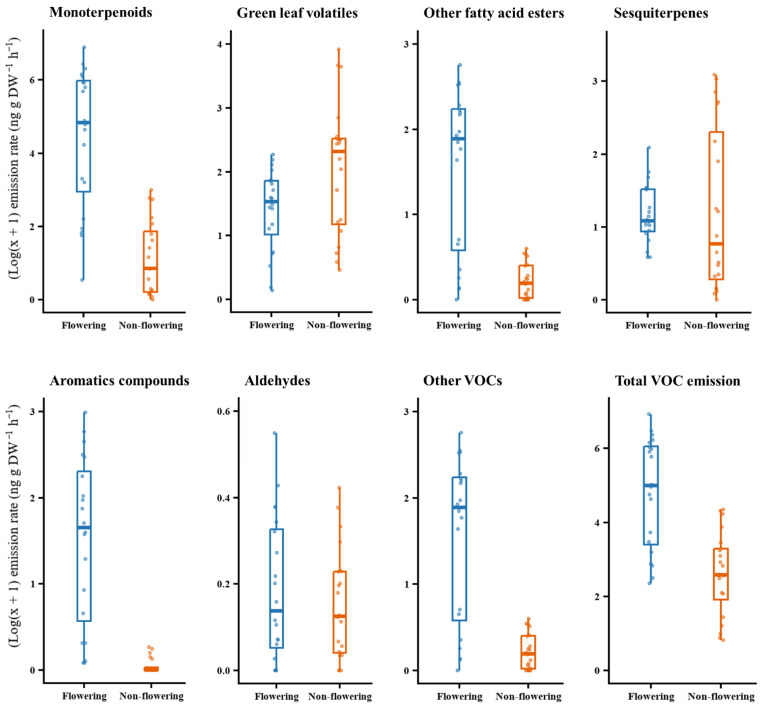
Comparisons of major chemical classes found in the headspace of Scotch broom (*n* = 20). Total VOC emission was calculated as the sum of the emission rates of individual compounds found in the headspace.

The compounds identified were grouped by their major chemical classes (Table 1), and their proportions were compared between flowering and non-flowering plants using the Wilcoxon rank-sum test. Overall, flowering plants emitted a significantly higher quantity of VOCs (*W* = 344, *p* < 0.001). The proportion of monoterpenoids (*W* = 368, *p* < 0.001), other fatty acid esters (*W* = 381, *p* < 0.001), aromatic compounds (W = 385, *p* < 0.001), and other VOCs (*W* = 347.5, *p* < 0.001) were significantly higher in flowering plants. Green leaf volatiles were significantly higher in non-flowering plants (*W* = 118, *p* = 0.027) while aldehydes (*W* = 217, *p* = 0.655) and sesquiterpenes (*W* = 228, *p* = 0.457) did not differ between the two phenological stages (Figure 4).

### 2.2. Arthropods on Scotch Broom

**Figure 5:**
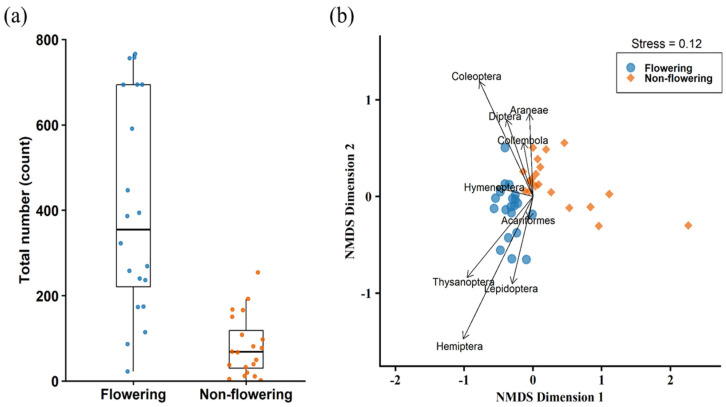
(**a**) Total number of arthropods on Scotch broom. (**b**) Arthropod community composition on flowering and non-flowering stages.

The total number of arthropods collected on flowering plants was significantly higher than that on non-flowering plants (Wilcoxon rank-sum test: *W* = 367, *p* < 0.001, (Figure 5a). There was also a significant difference between arthropod composition on flowering and non-flowering plants (PERMANOVA: Df = 1, pseudo-*F* = 14.69, *p* = 0.001). Araneae (*R*^2^ = 0.197, *p* = 0.013), Hemiptera (*R*^2^ = 0.856, *p* = 0.001), Coleoptera (*R*^2^ = 0.541, *p* = 0.001), Diptera (*R*^2^ = 0.209, *p* = 0.013), Thysanoptera (*R*^2^ = 0.435, *p* = 0.001), and Lepidoptera (*R*^2^ = 0.243, *p* = 0.005) were associated with patterns in community composition (Figure 5b). However, of these only Araneae, Hemiptera, and Thysanoptera differed significantly between the two phenological stages (Table 3).

### 2.3. Abiotic Factors

Temperature was significantly higher during late summer, when plants were not flowering, compared to the flowering season. Among soil properties, water content was significantly higher in the non-flowering season, whereas phosphorus was significantly higher during the flowering season, while % total N and potassium showed no significant difference (Table 4).

### 2.4. Association Between Biotic and Abiotic Factors and VOC Emissions

Biotic (Table 3) and abiotic (Table 4) factors that differed significantly between the flowering and non-flowering periods were selected as predictors. Their associations with the major chemical classes that also showed significant variation and total VOC emissions (shown in Figure 4) were examined. Results are shown in Table 5 and Table 6.

In summary, among the tested biotic factors, Araneae had significant negative associations with monoterpenoids, other fatty acid esters, and total VOC emissions. Hemiptera had significant negative association with green leaf volatiles but was positively associated with other fatty acid esters and aromatic compounds, while Thysanoptera had positive association with other fatty acid esters.

Regarding abiotic factors, temperature had a significant positive association with green leaf volatiles but was negatively associated with the remaining chemical classes and total VOC emissions. Soil water content (SWC) had no significant association with any of the tested response variables. Phosphorus had significant positive associations with monoterpenoids and total VOC emissions.

## 3. Discussion

Scotch broom produces tissue-specific volatile compounds [12,16,17,18] which are influenced by abiotic factors and linked with changes in arthropod communities, yet these factors have not been studied together in either its native or invaded range. Here we characterised the VOC emissions from Scotch broom as an invasive species, during the flowering and non-flowering stages, and established links between VOCs and biotic and abiotic factors, with the aims of (1) identifying floral vs. vegetative stage VOC components, (2) testing for shifts in arthropod abundance and functional composition between phenological phases, and (3) assessing potential environmental factors linked to chemical signalling in this invasive shrub. These data will inform mechanistic understanding of plant–arthropod interactions in this invaded system and could identify compounds of interest for further investigation to aid Scotch broom management.

Our data revealed significant differences between flowering and non-flowering Scotch broom in the Central Plateau of New Zealand’s North Island. Out of 56 compounds, over half were exclusively emitted by flowering plants, mainly monoterpenes, aromatics, and fatty acid esters (other than green leaf volatiles), suggesting that these are mostly floral compounds. In contrast, other compounds (mainly green leaf volatiles) were dominant in non-flowering Scotch broom and are likely associated with vegetative (leaf) tissue, as their name suggests. However, it is also possible that there are some changes in emissions from vegetative tissue between the two stages.

Plants that rely on insects for pollination invest considerable resources in the production of floral volatiles. Such volatiles are also likely to vary depending on the type of pollinator, with some plants releasing unique compounds to attract specialist pollinators [23,25,42,43]. Although Scotch broom can self-pollinate, it predominantly depends on cross-pollination for successful reproduction, thus it is not surprising that it releases a complex bouquet of VOCs to attract pollinators like bees and bumblebees, which are also present in its native range. Scotch broom flowers require triggering by a pollinator for the keel petals to open, allowing pollen to be deposited on the visiting insect [44,45]. Studies in other regions where this species is invasive suggest that floral traits are under selection pressure to enhance pollinator visitation [46].

Previous work on Scotch broom in its native range in Spain has shown that flowering tissue emits a characteristic suite of VOCs and that extracts or headspace collections from flowering plants can inhibit the germination and seedling growth of other plants, indicating the potential direct (phytotoxic/allelopathic) and indirect ecological effects of those volatiles [16,17,18,47]. Pardo-Muras and coworkers identified 28 volatile compounds from Scotch broom [17], about half of those identified here, and while this may be due to different methodologies or other reasons, this may suggest that this species is indeed under selective pressure to produce an abundant and diverse blend of VOCs in its invaded ranges to attract additional pollinators, especially since invasive plants can grow in dense clusters and in close proximity to native plants, so there may be both intra- and interspecific competition for pollinators. A study on 70 vascular plants from Hawaii, shows that alien species produce more VOCs in comparison to natives, suggesting this may be true for other invasive species as well [38].

Like Pardo-Muras et al. [17], we found abundant monoterpenes such as terpinen-4-ol, verbenol, *α*-terpineol, and verbenone in the headspace of Scotch broom, as well as other compounds not previously reported for this species. This suggests high metabolic diversity in Scotch broom, and while genetic factors are partly responsible for the observed differences, it is likely that this invasive plant also shows high phenotypic plasticity, contributing to its ecological success [48,49,50].

Floral VOCs are chemically and functionally distinct from vegetative emissions in many species: flowers typically emit blends tailored to attract particular pollinators (but can also repel flower feeders and non-mutualistic pollinators), whereas vegetative emissions may be more concerned with direct or indirect defence by repelling herbivores and attracting predators or parasitoids of these herbivores [24,25,43]. Consequently, the onset of flowering often changes the olfactory landscape of a plant at both short (within-plant) and landscape scales, with implications for the composition and behaviour of arthropod assemblages that interact with it [51,52,53].

Knowledge of the arthropod fauna associated with Scotch broom in New Zealand is important for both ecological understanding and biological control planning. Early surveys documented a diverse assemblage of phytophagous, pollinating and predatory arthropods using Scotch broom in New Zealand [54], with relatively more generalist plant-feeders and pollen-feeding taxa in the introduced range than the native range [55]. Recent reports support earlier findings showing that invasive Scotch broom attracts a wide variety of native and introduced arthropods, often having higher arthropod diversity and abundance than native plants [9,11].

In this study, we found that arthropod communities varied significantly between phenological stages, with Hemiptera, Thysanoptera and Lepidoptera being more abundant in flowering plants. Among the Hemiptera, the introduced broom psyllid (*Arytainilla spartiophila*) was particularly abundant, suggesting that while the plant may be aiming to attract potential pollinators it is also attracting this specialist biocontrol agent which could promote biocontrol efforts. Thrips, on the other hand, feed on soft plant tissue like flower petals, buds, and pollen, and although causing some harm to the plant, can also act as pollinators. Similarly, Lepidoptera, especially adults, are general pollinators, while larvae may feed on shoots, leaves, and other tissue. This suggests that there must be a trade-off between reproduction and defence [56,57,58].

The strong correlation between fatty acid esters and plant-feeding Hemiptera and Tysanoptera suggests that some of these compounds may be involved in host plant selection by these groups. The Myrid bug *Apolygus lucorum* (Hemiptera) is known to use fatty acid esters to detect flowering from non-flowering plants [59], supporting this hypothesis. Furthermore, it has been shown that trips also use volatiles to select their host plants, showing a preference for flowering stages containing fatty acid esters [31,60]. While these compounds may not be the most abundant in the floral bouquet of Scotch broom, the insect olfactory system is exquisitely sensitive, and minor compounds are known to mediate highly specific plant–insect interactions [61]. Further exploration of these compounds may be valuable to support biocontrol efforts using *A. spartiophila.*

Green leaf volatiles are defence compounds typically released from leaf tissue almost immediately after mechanical damage; thus, they are excellent sources of information for predators and parasitoids of herbivores, which use them as cues to find potential prey [62,63,64]. Therefore, it is likely that during non-flowering stages, plants will invest more resources in the production of these defensive compounds, as reproduction is no longer a priority. For this reason, it is not surprising that the presence of spiders (Araneae)—which are generalist predators—is highly correlated with non-flowering plants and the emission of green leaf volatiles. While chemical communication in spiders is still poorly understood, they are known to use chemical signals to interact with conspecifics and their environment [65,66,67]. In the case of Hemiptera, including the broom psyllid, a negative correlation with green leaf volatiles may indicate that they avoid non-flowering plants. Possible reasons for avoidance include direct toxicity or deterrent effects, the information value associated with inedible hard leaf tissue, or higher risk of predation. However, it is important to note that other cues (visual and tactile) also play a role in pollinator, herbivore, and predator preferences.

The last variable in this equation is the association between environmental factors and VOC emissions. Temperature is consistently reported as a key factor modulating VOCs. The impact of temperature on VOCs may be related to enzymatic activity, stomatal opening, cell membrane structure and fluidity, or its direct impacts on compound volatility, diffusion, and stability. Generally, higher temperatures favour VOC production and release, up to an optimal point, which may differ depending on the species and its phenology, with low and high temperature extremes typically having limiting effects [68,69,70]. Therefore, it is unsurprising that temperature is significantly correlated (either positively or negatively) with the emission of all tested compound classes. Green leaf volatiles are small molecules that are released from membrane breakage, and increased temperatures can impact membrane integrity and enzymatic activities, leading to higher emissions [64,70,71]. For the remaining groups of compounds, especially for terpenoids, production and emission are regulated differently, as these are synthesized in different plant parts or tissues, can be mobilised and stored, and require light-dependent reactions, making temperature-related effects more complex [71,72,73]. Further experiments under controlled conditions are needed to determine the temperature-dependent emissions of different compound classes.

Phosphorus also seemed to have an important impact on monoterpenes and total VOC emissions. While the role of phosphorus on VOC emissions is less clear than that of temperature, some studies on cyanobacteria show that different phosphorus sources and concentrations can impact VOC emissions, leading to different odour blends [74,75], so it is likely that this is also true for plants. The role of phosphorus remains to be further investigated. While soil water content differences were observed, these were not linked to VOC emissions under our experimental conditions, but it is expected that water stress (drought or flooding) would cause an impact, as observed in other systems [76,77,78].

This work contributes to understanding how VOC profiles change between flowering and non-flowering stages and how these changes are associated with biotic and abiotic factors. By linking VOC emission patterns across phenological stages to pollinator activity, herbivory, and natural enemy attraction, this study provides insights that can guide future research into management or biocontrol strategies aimed at limiting the reproduction and spread of this invasive shrub. As this study was conducted under natural conditions, variability in VOC emissions may also be associated with other factors not identified in this study.

Future research should (1) quantify VOC emissions using larger sample sizes in both native and introduced ranges to assess whether observed associations between VOCs, arthropods, and abiotic factors are similar across these ranges; (2) use controlled experiments to further investigate how abiotic factors influence VOC emissions of this species, e.g., by growing plants under different temperature and soil nutrient regimes; and (3) directly test the role of specific compounds that vary between phenological states (Table 2 and Table S1) in mediating interactions between Scotch broom and other organisms such as pollinators, herbivores, natural enemies, and neighbouring plants.

## 4. Materials and Methods

### 4.1. Site Description

The study was conducted under natural conditions on the Central Plateau of the North Island, New Zealand, during summer 2017. Four sites with Scotch broom present were selected for sampling. Three sites were within the Waiouru Military Training Area (WMTA) and the fourth was near Erua, on the western boundary of Tongariro National Park. WMTA site coordinates are as follows: site one Long. 175.737467–Lat. −39.315117, site two Long. 175.732783–Lat. −39.3142, site three Long. 175.3907–Lat. −39.244033, and at Erua, site four Long. 175.737–Lat. −39.315383. At each site, we selected five similar-sized broom plants, providing a total of 20 plants, from which we measured aboveground VOCs emission and recorded arthropods on the foliage.

**Figure 6:**
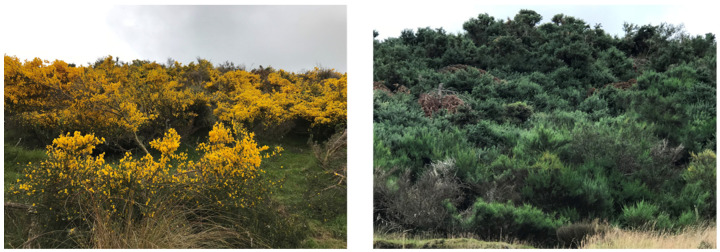
Scotch broom plants within the Waiouru Military Training Area, showing flowering and non-flowering stages. Photos taken by Evans Effah.

Ambient temperature measurements and soil analyses were also conducted at each site following the protocols described in [79]. Data were collected from the same plants in early summer (December 2017), when they were flowering profusely, and repeated in late summer (February 2018), when flowers were absent and the plants setting seeds, representing distinct phenological stages (Figure 6).

### 4.2. Measuring VOC Emissions

Volatile organic compounds were collected for 2 h using a portable PVAS22 pump (Volatile Assay Systems, Rensselaer, NY, USA), from similar amounts of foliage enclosed in new oven bags (Glad^®^, Oakland, CA, USA). Carbon-filtered air was pushed in (1.70 L/min) and pulled out (1.20 L/min) and trapped on 30 mg HayeSep Q adsorbent filters. The foliage was then excised and oven-dried at 60 °C for 72 h to calculate emissions per dry weight (DW). Sampling was conducted simultaneously across sites to minimise environmental variability.

Trapped VOCs were eluted from filters using 200 µL of 95% hexane containing 10 ng mL^−1^ nonyl acetate (Sigma Aldrich, Buchs, Switzerland) as an internal standard. Samples were analysed using gas chromatography–mass spectrometry (QP2010; GCMS Solution v2.70, Shimadzu Corporation, Kyoto, Japan) with a 30 m × 250 μm × 0.25 μm TG-5MS capillary column (Thermo Fisher Scientific, Waltham, MA, USA). Helium served as the carrier gas at 53.5 kPa, with a total flow of 14.0 mL/min, linear velocity of 36.3 cm/s, and purge flow of 3.0 mL/min. The oven temperature was set to 50 °C and held for 3 min, increased to 95 °C at 5 °C/min, and then ramped to 230 °C. Chromatographic analyses were conducted in scan mode, with peaks quantified relative to the internal standard. Emission rates were then normalised by the DW of foliage and sampling duration (hours) to calculate emissions in DW h^−1^. Compounds were tentatively identified by comparison with spectra in the National Institute of Standards and Technology MS library and, where available, confirmed using authentic standards from Sigma-Aldrich.

### 4.3. Arthropod Community Composition

Arthropods were collected from each plant using the beating tray method immediately after volatile sampling on a branch adjacent to the one used for VOC collection. Foliage was beaten three times over a white plastic tray, and the specimens were preserved in 70% ethanol and identified to order [9].

### 4.4. Ambient Temperature and Soil Analyses

Ambient air temperature was recorded using Tinytag^®^ (TGP-4500, Gemini) data loggers protected by Stevenson Type Screens (Hastings Data Loggers, Port Macquarie, NSW, Australia), installed 50 cm above ground at each site, beginning 10 days before VOC measurements and retrieved on the final day of measurements each season [10]. Loggers were programmed to record temperature every 30 min.

Soil properties were assessed by homogenising 20 cores (15 cm × 3 cm) per site to obtain a representative mean value for each site. Soil moisture content was measured gravimetrically (%), and total nitrogen (N), available phosphorus (P), and potassium (K) were analysed by a commercial laboratory (Hills Laboratories, Hamilton, New Zealand).

### 4.5. Data Analyses

All statistical analyses were conducted in R (version 4.5.1). Major volatile classes, number of compounds, temperature, soil properties, and total arthropod counts on Scotch broom were compared between flowering and non-flowering periods using the non-parametric Wilcoxon rank-sum test and data were visualised with boxplots.

Arthropod community composition on flowering and non-flowering Scotch broom plants was assessed using permutational multivariate analysis of variance (PERMANOVA) in the vegan package [80], based on Bray–Curtis dissimilarities after log10(x + 1) transformation. Non-metric multidimensional scaling (NMDS), also based on Bray–Curtis dissimilarities, was used to visualise variation in community composition, and the envfit function was applied to identify arthropod groups significantly associated with ordination patterns.

We used random forest analysis to classify the volatile blends of Scotch broom during flowering and non-flowering periods, and a multidimensional scaling (MDS) plot was used to visualise sample clustering [81]. The ten VOCs that contributed most to the separation of blends were identified, and their abundances were compared between flowering and non-flowering plants using the Wilcoxon rank-sum test.

We then examined associations between the ten VOCs and biotic and abiotic factors by using generalised linear models with Tweedie distribution (link = log), which handles positive, right-skewed data with zeros. The model was fitted using the glmmTMB package [82]. VOC classes were treated as response variables, and all predictors were standardised. To reduce the risk of overfitting given the small sample size and large number of predictors, separate models were fitted for biotic and abiotic factors. Because loggers recorded data every 30 min, resulting in thousands of observations, temperature data were aggregated to the site level using mean values for each site.

## Figures and Tables

**Table 1:** Compounds found in the headspace of Scotch broom from both flowering and non-flowering plants.

Code	Compound	Code	Compound	Code	Compound
	**Monoterpenoids**		**Green leaf volatiles**		**Aromatics**
EBO	(*E*)-β-Ocimene	Z3H	(*Z*)-3-Hexenol	PAL	Phenylethyl alcohol
2PINOL	2-Pinen-10-ol	Z3HA	(*Z*)-3-Hexenyl acetate	MSAL	Methyl salicylate
2CAR	2-Carene	E2H	(*E*)-2-Hexanal	BENA	Benzaldehyde
ZBO	(*Z*)-β-Ocimene	HEX	Hexanol	BAC	Benzyl acetate
CPIN	Cis-pinen-3-ol	Z2H	(*Z*)-2-Hexenol	BAL	Benzyl alcohol
CHR	Chrysanthenone	Z2HA	(*Z*)-2-Hexenyl acetate		
CAR	Carveol	HAC	Hexyl acetate		**Other VOCs**
CAM	Camphene			2NOR	2-Norpinanone
E3C	(*E*)-3(10)-Caren-4-ol		**Other fatty acid esters**	2UND	2-Undecanone
LAC	Linalool acetate	Z3H2M	(*Z*)-3-Hexenyl 2-methylbutanoate	3MBA	3-Methyl-1-butanol acetate
NER	Nerol	H2MB	Hexyl 2-methylbutanoate	EOCT	Ethyl octanoate
EUC	Eucalyptol	Z3HB	(*Z*)-3-Hexenyl butyrate	E2OC	(*E*)-2-Octenol
pCym	p-Cymene	EHEX	Ethyl hexanoate		
EUG	Eugenol acetate				
SAB	Sabinene		**Sesquiterpenes**		
SYL	Sylvestrene	aBOU	α-Bourbonene		
TER4	Terpinen-4-ol	ZAF	(*Z*,*E*)-α-Farnesene		
TER	Terpinolene	EAF	(*E*,*E*)-α-Farnesene		
VER	Verbenone	EBC	(*E*)-β-Caryophylene		
aPHE	α-Phellandrene	COP	Copaene		
pain	α-Pinene	HUM	Humulene		
aTER	α-Terpineol	aPAN	α-Panasinsene		
aTHU	α-Thujene				
bMYR	β-Myrcene		**Aldehydes**		
bPIN	β-Pinene	DEC	Decanal		
gTER	γ-Terpinene	NON	Nonanal		

**Table 2:** Comparison of top ten compounds with high contributions in distinguishing the blends of flowering and non-flowering Scotch broom plants (*n* = 20). Wilcoxon rank-sum test. *p*-value less than 0.05 was considered significant.

	Median (IQR) (ng g DW^−1^ h^−1^)		Wilcoxon Test
Compound	Flowering	Non-Flowering	*p*-Value
α-Pinene	67.06 (190.82)	0.00 (0.20)	<0.001
α-Phellandrene	13.09 (36.97)	0.00 (0.00)	<0.001
Sabinene	22.95 (78.37)	0.00 (0.00)	<0.001
Sylvestrene	5.75 (15.24)	0.00 (0.00)	<0.001
Verbenone	6.36 (18.40)	0.00 (0.00)	<0.001
Terpinen-4-ol	1.75 (2.52)	0.00 (0.00)	<0.001
Ethyl hexanoate	2.31 (1.16)	0.00 (0.00)	<0.001
Hexyl 2-methylbutanoate	0.39 (0.23)	0.00 (0.00)	<0.001
Benzyl acetate	3.57 (7.90)	0.00 (0.00)	<0.001
2-Undecanone	2.83 (6.57)	0.00 (0.00)	<0.001

**Table 3:** Comparison of arthropod abundance on flowering and non-flowering Scotch broom plants. Wilcoxon rank-sum test. *p*-value less than 0.05 was considered significant.

	Count (Median (IQR))		Wilcoxon Test
	Flowering	Non-Flowering	*p*-Value
Araneae	2.00 (4.25)	5.50 (5.00)	0.017
Hemiptera	167.50 (372.25)	5.00 (7.25)	<0.001
Coleoptera	51.00 (96.75)	32.50 (69.75)	0.473
Diptera	1.00 (2.00)	1.00 (1.25)	0.796
Thysanoptera	16.50 (28.00)	1.00 (2.25)	<0.001
Lepidoptera	0.00 (1.00)	0.00 (0.00)	0.227
Hymenoptera	1.00 (1.00)	0.00 (1.00)	0.325
Collembola	0.00 (0.00)	0.00 (1.00)	0.138
Acariformes	5.50 (11.00)	7.00 (16.25)	0.249

**Table 4:** Comparison of measured abiotic factors between sampling periods. *p*-value less than 0.05 was considered significant.

	Measure (Median (IQR))		Wilcoxon Test
	Flowering	Non-Flowering	*p*-Value
Temperature (°C)	13.87 (8.17)	15.40 (7.00)	<0.001
Soil water content (%)	35.59 (12.44)	45.12 (12.79)	0.011
Total N (%)	0.30 (0.09)	0.32 (0.10)	0.999
Phosphorus (mg/L)	4.50 (2.00)	3.00 (1.00)	0.002
Potassium (me/100 g)	0.25 (0.06)	0.22 (0.10)	0.999

**Table 5:** Summary of GLM results for associations between biotic factors and VOC emissions.

Compound	Predictor	β	SE	*Z*	*p*-Value
Monoterpenoids	Araneae	−0.976	0.306	−3.189	0.001
	Hemiptera	0.179	0.336	0.534	0.593
	Thysanoptera	0.386	0.382	1.013	0.311
Green leaf volatiles	Araneae	0.065	0.168	0.390	0.697
	Hemiptera	−0.427	0.172	−2.487	0.013
	Thysanoptera	−0.210	0.154	−1.369	0.171
Other fatty acid esters	Araneae	−0.434	0.212	−2.042	0.041
	Hemiptera	0.339	0.160	2.111	0.035
	Thysanoptera	0.331	0.143	2.318	0.021
Aromatic compounds	Araneae	−0.548	0.325	−1.686	0.092
	Hemiptera	0.639	0.231	2.767	0.006
	Thysanoptera	0.396	0.227	1.746	0.081
Total VOC emissions	Araneae	−0.621	0.216	−2.872	0.004
	Hemiptera	0.138	0.265	0.519	0.604
	Thysanoptera	0.228	0.284	0.802	0.423

**Table 6:** Summary of GLM results for associations between abiotic factors and VOC emissions.

Compound	Predictor	β	SE	*Z*	*p*-Value
Monoterpenoids	Temperature	−1.296	0.215	−6.042	<0.001
	SWC	−0.292	0.204	−1.430	0.153
	Phosphorus	0.750	0.240	3.124	0.002
Green leaf volatiles	Temperature	0.558	0.213	2.616	0.009
	SWC	0.018	0.163	0.110	0.912
	Phosphorus	0.085	0.235	0.360	0.719
Other fatty acid esters	Temperature	−1.029	0.217	−4.748	<0.001
	SWC	0.008	0.155	0.050	0.960
	Phosphorus	0.119	0.178	0.668	0.504
Aromatic compounds	Temperature	−1.794	0.428	−4.196	<0.001
	SWC	−0.426	0.245	−1.742	0.082
	Phosphorus	0.077	0.303	0.255	0.799
Total VOC emission	Temperature	−0.675	0.177	−3.809	<0.001
	SWC	−0.217	0.151	−1.444	0.149
	Phosphorus	0.717	0.205	3.500	<0.001

## Data Availability

Data can be provided upon request.

## Supplementary Materials

The following supporting information can be downloaded at https://www.mdpi.com/article/10.3390/plants15010095/s1, Table S1: List of VOCs identified from the headspace of flowering and non-flowering Scotch broom plants, showing mean emissions and standard error.

## Abbreviations

The following abbreviations are used in this manuscript:

GC-MS	Gas chromatography and mass spectrometry
MSD	Multidimensional scaling
NMSD	Non-metric multidimensional scaling
PERMANOVA	Permutational analysis of variance
SWC	Soil water content
VOC(s)	Volatile organic compound(s)
