# Supplementary material for: Volatile Organic Compound Emissions in the Invasive Legume Cytisus scoparius: Linking Plant Phenology, Arthropod Communities, and Environmental Factors
Source: Plants (Basel). 2025 Dec 28;15(1):95. doi: 10.3390/plants15010095 (PMC12787757; doi:10.3390/plants15010095)
Supplement: Supplementary file 1 [file plants-15-00095-s001.zip › plants-4037420-supplementary.pdf]

Supplementary material

Table S1. List of VOCs identified from the headspace of flowering and non-flowering Scotch broom plants, showing mean emissions and standard error (ng × g × DW^{-1} × h^{-1}).

**Phenology**	**Flowering**<br><br>Mean ± SEM	**Non-flowering**<br><br>Mean ± SEM
(*Z*)-3-Hexenol	0.61 ± 0.13	2.22 ± 0.74
(*Z*)-3-Hexenyl 2-methylbutanoate	0.02 ± 0.01	0.15 ± 0.11
(*Z*)-3-Hexenyl acetate	3.08 ± 0.48	8.38 ± 2.07
(*E*)-2-Hexanal	ND	0.13 ± 0.04
(*E*)-2-Octenol	ND	0.28 ± 0.06
Hexanol	0.11 ± 0.04	0.22 ± 0.07
Hexyl 2-methylbutanoate	0.43 ± 0.05	ND
Ethyl hexanoate	2.45 ± 0.23	ND
Ethyl octanoate	0.04 ± 0.01	ND
(*Z*)-2-Hexenol	ND	0.43 ± 0.2
(*Z*)-2-Hexenyl acetate	ND	0.26 ± 0.14
(*Z*)-3-Hexenyl butyrate	ND	0.19 ± 0.1
Hexyl acetate	ND	0.19 ± 0.11
2-Norpinanone	0.98 ± 0.2	ND
2-Undecanone	3.97 ± 0.9	ND
3-Methyl-1-butanol acetate	0.59 ± 0.22	ND
Benzaldehyde	0.23 ± 0.06	ND
Benzyl acetate	4.84 ± 1.1	ND
Benzyl alcohol	0.26 ± 0.07	ND
Decanal	0.13 ± 0.03	0.08 ± 0.03
Nonanal	0.09 ± 0.03	0.09 ± 0.02
Phenylethyl alcohol	0.38 ± 0.2	ND
Methyl salicylate	ND	0.06 ± 0.02
α-Bourbonene	ND	0.17 ± 0.07
(*Z*,*E*)-α-Farnesene	ND	1.22 ± 0.49
(*E*,*E*)-α-Farnesene	0.63 ± 0.22	2.26 ± 0.81
(*E*)-β-Caryophylene	1.07 ± 0.11	1.43 ± 0.4
Copaene	0.15 ± 0.05	0.11 ± 0.04
Humulene	0.27 ± 0.07	0.21 ± 0.06
α-Panasinsene	0.41 ± 0.15	ND
(*E*)-β-Ocimene	0.06 ± 0.02	0.19 ± 0.06
2-Pinen-10-ol	0.15 ± 0.07	ND
2-Carene	0.29 ± 0.07	ND
(*Z*)-β-Ocimene	1.16 ± 0.32	3.7 ± 1.2
Cis-pinen-3-ol	0.52 ± 0.13	ND
Chrysanthenone	0.98 ± 0.19	ND
Carveol	0.77 ± 0.19	ND

Camphene	0.24 ± 0.05	ND
(*E*)-3(10)-Caren-4-ol	0.24 ± 0.08	ND
Linalool acetate	2.34 ± 0.51	ND
Nerol	1.4 ± 0.33	ND
Eucalyptol	0.72 ± 0.17	ND
p-Cymene	0.12 ± 0.04	ND
Eugenol acetate	0.1 ± 0.05	ND
Sabinene	47.11 ± 11.89	ND
Sylvestrene	9.52 ± 2.29	ND
Terpinen-4-ol	1.91 ± 0.39	ND
Terpinolene	1.02 ± 0.27	ND
Verbenone	12.62 ± 3.08	ND
α-Phellandrene	26.11 ± 7.15	ND
α-Pinene	123.54 ± 33.41	0.16 ± 0.06
α-Terpineol	0.25 ± 0.07	ND
α-Thujene	0.81 ± 0.2	ND
β-Myrcene	0.42 ± 0.09	ND
β-Pinene	11.21 ± 2.64	0.11 ± 0.04
γ-Terpinene	2.42 ± 0.47	ND

ND = not detected
